# Focusing on Rare Variants Related to Maturity-Onset Diabetes of the Young in Children

**DOI:** 10.1155/pedi/8155443

**Published:** 2025-01-28

**Authors:** Yu Ding, Qianwen Zhang, Shiyang Gao, Juan Li, Guoying Chang, Yirou Wang, Libo Wang, Xin Li, Yao Chen, Ru-en Yao, Tingting Yu, Niu Li, Dan Lou, Xiumin Wang

**Affiliations:** ^1^Department of Endocrinology and Metabolism, Shanghai Children's Medical Center, Shanghai Jiaotong University School of Medicine, Shanghai 200127, China; ^2^Department of Medical Genetics and Molecular Diagnostic Laboratory, Shanghai Children's Medical Center, Shanghai Jiaotong University School of Medicine, Shanghai 200127, China; ^3^Department of Reproductive Genetics, International Peace Maternity and Child Health Hospital, Shanghai Jiao Tong University School of Medicine, Shanghai 200030, China; ^4^Department of Pediatrics, The First Affiliated Hospital of Henan University of Science and Technology, Henan University of Science and Technology, Luoyang 471000, China

**Keywords:** childhood, MODY, next-generation sequencing, rare subtypes

## Abstract

**Background:** In this study, we analysed the clinical and genetic characteristics and follow-up data of patients with maturity-onset diabetes of the young (MODY).

**Methods:** From January 2015 to December 2022, patients with persistent hyperglycaemia suspected of having monogenic diabetes or diabetes syndrome were recruited, and next-generation sequencing (NGS) was performed at the Shanghai Children's Medical Center. Patients' clinical and laboratory findings were recorded preceding follow-ups. Candidate variants were verified using Sanger sequencing. Variant pathogenicity was evaluated according to the American College of Medical Genetics and Genomics (ACMG) guidelines.

**Results:** Genetic testing was performed in 175 children. MODY-related pathogenic or likely pathogenic gene variants were identified in 30 patients from different families. Of these, 11 were diagnosed with *GCK*-MODY (36.7%), six with *INS*-MODY (20%), five with *HNF1A*-MODY (16.7%), five with *ABCC8*-MODY (16.7%), two with *HNF1B*-MODY (6.7%) and one with *HNF4A*-MODY (3.3%). There was one shift variant and seven splice-site variants, and the rest were missense variants. We discovered six novel variants. Of the 30 patients, 63.3% had a family history of diabetes, 13.3% had diabetic ketoacidosis (DKA), and 16.7% had positive diabetes-associated autoantibodies. The diabetes phenotype of patients with the *INS* variant was similar to that of patients with type 1 diabetes. All patients, including those having positive autoantibodies, required long-term insulin therapy during follow-ups. Four patients with the *ABCC8* variant were unable to switch to oral sulfonylurea therapy and continued insulin therapy.

**Conclusion:** Genetic testing is helpful for the precise diagnosis and treatment of patients with MODY, including those with DKA history and positive diabetes autoantibody. *GCK*-MODY is the most common type of MODY, and patients with *INS* variant account for a relatively large proportion of MODY cases in our cohort.

## 1. Introduction

The age of diabetes onset has an important relationship with its classification. The earlier the age of onset, the higher is the probability of having a genetic predisposition for the disease. Therefore, greater priority should be given to recognizing the risk of monogenic diabetes and related syndromes in children and adolescents with diabetes. Maturity-onset diabetes of the young (MODY) is the most prevalent form of monogenic diabetes with partly preserved pancreatic *β*-cell function [1]. It typically manifests as an autosomal dominant mode of inheritance, and the age of onset is usually less than 25 years [2]. Currently, MODY is classified into 14 subtypes, each of which is caused by variants in different genes. The most common MODY subtypes include *GCK*-(MODY2), *HNF1A*-(MODY3), *HNF4A*-(MODY3) and *HNF1B*-(MODY5), accounting for over 80% of all MODY cases [3, 4]. Other subtypes, including *IPF1/PDX1*-(MODY4), *NEUROD1*-(MODY6), *KLF11*-(MODY7), *CEL*-(MODY8), *PAX4*-(MODY9), *INS*-(MODY10), *BLK*-(MODY11), *ABCC8*-(MODY12), *KCNJ11*-(MODY13) and *APPL1*-(MODY14), are considered to be the rarer subtypes [5, 6]. While common subtypes of MODY have been thoroughly researched and understood, little is known about the rarer subtypes.

*GCK*, *HNF4A* and *HNF1A* are the most common types of MODY in China [7–9]. With the development of genetic detection technology, reduction in cost and improvement in accuracy, next-generation sequencing (NGS) has led researchers to finding more gene variations related to rarer MODY subtypes in children with diabetes or persistent hyperglycaemia and improving diagnosis accuracy and treatment [10]. However, owing to differences in genetic backgrounds among different countries and races, the incidence rate and genetic variability of rare types of MODY are high [11, 12]. To the best of our knowledge, there is a lack of research on the genetic spectrum of rare MODY subtypes in Chinese children. Some rare MODY subtypes have been reported as individual cases, with few case series and follow-up data, making it difficult to obtain detailed clinical data. And all these factors limit the in-depth understanding of these diseases.

Genetic testing was performed on children with diabetes or persistent hyperglycaemia at our centre, and MODY-related genetic variations were found. Among them, the proportion of rare MODY variants, *INS* and *ABCC8*, was high. In this study, we analysed the clinical and genetic characteristics and follow-up data of these patients to enable clinicians to better understand these MODY subtypes so that they may provide precise treatment.

## 2. Methods

### 2.1. Participants

**Figure 1:**
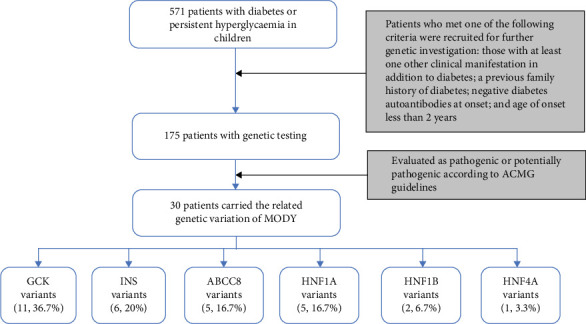
The flow chart of patient recruitment. Among the 571 patients, genetic investigations were performed in 175 patients who met the inclusion criteria. Thirty patients with maturity-onset diabetes of the young (MODY) from different families were identified and included in the final analysis.

Between January 2015 and December 2022, a total of 571 patients under 18 years of age were diagnosed with diabetes or persistent hyperglycaemia, excluding drug-induced or secondary diabetes at the Shanghai Children's Medical Centre. Patients who met one of the following criteria were recruited into the study for further genetic testing: those with at least one other clinical manifestation in addition to diabetes, including the nervous, digestive, cardiovascular, urinary and immune systems; a previous family history of diabetes; negative diabetes autoantibodies at onset; and age of onset less than 2 years. Hence, a total of 175 patients were included (Figure 1).

This study was conducted in accordance with the Declaration of Helsinki and was approved by the Ethics Committee of Shanghai Children's Medical Center (Shanghai, China). All blood samples were analysed after obtaining informed consent from the patients' parents.

All of the patients' information including sex, age at diagnosis, weight (kg), height (cm), body mass index (BMI, kg/m^2^), fasting glucose level, fasting C-peptide level (ng/mL), glycosylated haemoglobin (HbA1C) level, presence of diabetes autoantibodies (islet cell antibodies [ICA], tyrosine phosphatase antibodies [IA-2A], insulin autoantibodies [IAA] and glutamic acid decarboxylase antibodies [GADA]) at initial diagnosis, treatment method (oral antidiabetic drug, insulin) and follow-up process were recorded in digital medical system. In addition, some patients underwent oral glucose tolerance testing (OGTT).

### 2.2. Molecular Genetic Analysis

Targeted gene panel sequencing (TGS) was performed as previously described [13], and 51 patients underwent TGS before 2019. The remaining patients underwent whole-exome sequencing (WES). The adjustment of the detection method was due to the update of the genetic testing method in our hospital, and a more comprehensive testing method (WES) has been carried out for genetic testing since 2019. The Inherited Disease Panel Kit (including 2742 genes) probe was selected to capture the exon and lateral intron regions of the target gene. The SureSelectXT Human All Exon Kit v6 (Agilent Technologies, Santa Clara, CA, USA) was used to enrich the coding exons and flanking intronic regions. Sequencing data were analysed using NextGENe software and the Ingenuity online software system.

Sanger gene sequencing was performed to validate and identify the origin of the candidate variants by specific primers designed using the UCSC ExonPrimer online software (http://genome.ucsc.edu/index.html). The pathogenicity of the variants was categorized according to the American College of Medical Genetics and Genomics (ACMG) guidelines [14] and further refined based on the ClinGen Sequence Variant Interpretation Working Group (SVI WG) (https://www.clinicalgenome.org/working-groups/sequence-variant-interpretation/) [15, 16]. The genetic diagnosis was made when variants were categorized as likely pathogenic or pathogenic and conformed to clinical manifestations.

### 2.3. Statistical Analysis

The statistical analysis was performed by using Stata 13.0 (Stata Corporation, College Station, TX, USA). Student's *t*-tests were used to measure the continuous variables of intergroup participant characteristics. The Pearson chi-square test was performed to compare intergroup categorical variables. If *p* values were less than 0.05, difference was considered statistically significant.

## 3. Results

### 3.1. Identification of Variants and Evaluation of Pathogenicity

**Figure 2:**
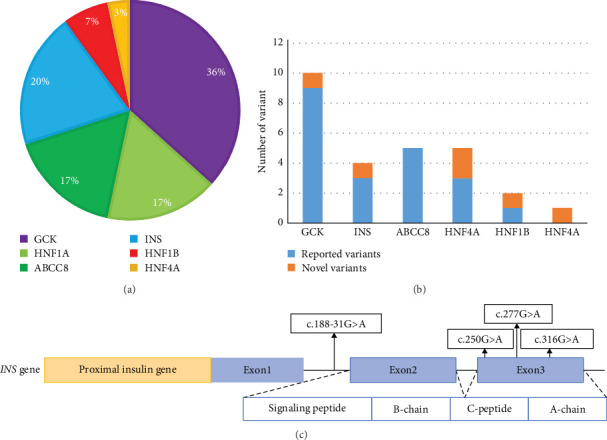
Genetic characteristics of patients with MODY. (A) Distribution of detected gene variants associated with MODY. (B) Distribution of the variants newly discovered in our cohort. (C) Location of the variants found in patients with *INS*-MODY. MODY, maturity-onset diabetes of the young.

Totally, 30 variants in six MODY-related genes were identified. Pathogenicity was classified as likely pathogenic or pathogenic variants base on the standard criteria recommended by the ACMG guidelines in 30 patients from 30 families (Figure 1, Table 1): 11 patients carried *GCK* variants, six carried *INS* variants, five carried *ABCC8* variants, five carried *HNF1A* variants, two carried *HNF1B* variants, and one carried the *HNF4A* variant. All the variants were heterozygous (Figure 2A). There was one shift variant and seven splice-site variants, and the rest were missense variants. Six variants were novel, not included in databases, such as HGMD and gnomAD (Figure 2B). Four variant sites were found in six *INS*-MODY cases, except for one splice variant, whereas the other three variants were located in the third exon region of the gene (Figure 2C).

### 3.2. Clinical Characteristics of Patients With MODY

**Figure 3:**
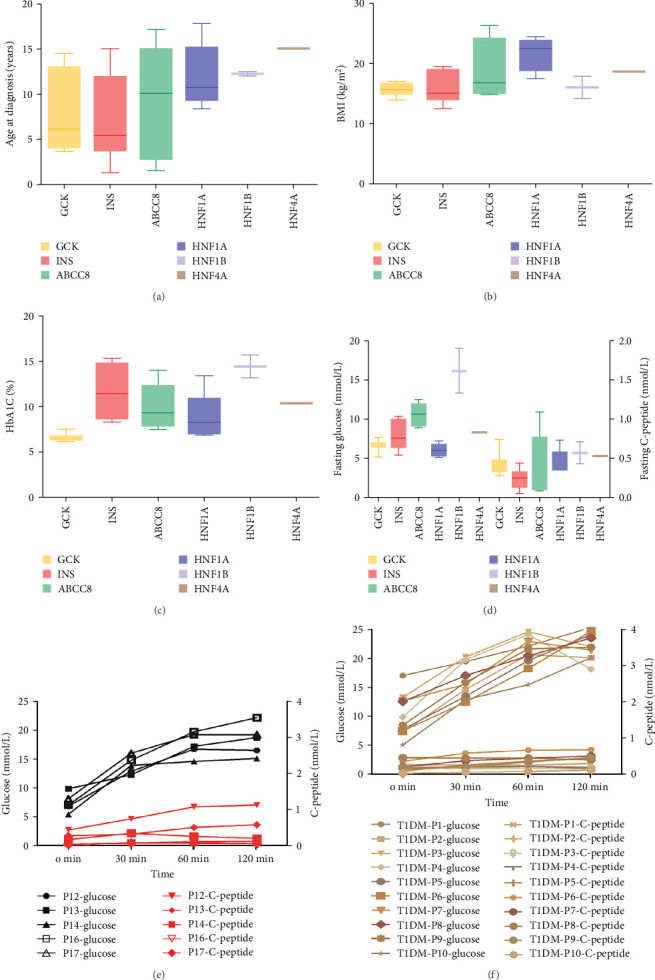
Clinical characteristics of patients with MODY. (A) Distribution of diagnosis age. (B) Distribution of BMI. (C) Distribution of HbA1c. (D) Distribution of fasting glucose and C-peptide. (E) OGTT of five patients with *INS*-MODY. (F) OGTT of 10 patients with T1DM with diabetes autoantibody positive. BMI, body mass index; HbA1C, glycosylated haemoglobin; MODY, maturity-onset diabetes of the young; OGTT, oral glucose tolerance testing; T1DM, type 1 diabetes mellitus.

Thirty patients were accurately diagnosed based on the genetic testing results combined with clinical manifestations (Table 2), half of whom were male. The age of patients at diagnosis was 9.19 ± 4.79 years (mean ± standard deviation), and the youngest average age was 7.01 in patients with *INS* variants (Figure 3A) in which four out of six (66.7%) of the patients were under 6 years old (Appendix Table S1). Nineteen patients (63.3%) had a family history of diabetes, including all patients with *GCK* or *HNF4A*/*1A* variants. There were six patients with the *INS* variant, four with the *ABCC8* variant and one with the *HNF1B* variant without a family history of diabetes. The variants identified in seven patients were de novo (five with the *INS* variant, one with the *ABCC8* variant and one with the *HNF1B* variant). The *INS* variant carried by Patient 15 came from patient's father, and the four *ABCC8* variants (Patients 19–22) were related to their father or mother; however, the parents did not have diabetes when their child was diagnosed. BMI was 17.37 ± 3.38 kg/m^2^ and significantly higher among patients with the *ABCC8* or *HNF4A*/*1A* variants (Figure 3B). The mean level of HbA1c at diagnosis was 9.16 ± 3.04% and higher among patients with the *INS* or *HNF1B* variants (Figure 3C). The fluctuations in fasting blood glucose and C-peptide levels were relatively large in these patients (Figure 3D). There was one patient with the *INS* variant, two with the *ABCC8* variant and one with the *HNF1B* variant who had diabetic ketoacidosis (DKA) at initial diagnosis. Antibodies against diabetes were found in five patients (16.7%). All patients required medication to control their blood sugar levels except for patients with *GCK* variants. All patients with the *INS* variant and *HNF1B* variant and 80% of patients with the *ABCC8* variant were treated with insulin.

Among the five patients with the *HNF1A*-MODY variant, four were adjusted to sulfonylurea therapy, while one struggled with poor blood sugar control and continued to use insulin therapy. Another patient with the *HNF1A*-MODY had good blood sugar control after long-term metformin and insulin therapy before diagnosis and was therefore not adjusted to sulfonylurea therapy for family factors. One patient with *HNF4A*-MODY was treated with sulfonylurea and metformin, and blood glucose control was good after 1 year of follow-up. At the last follow-up, HbA1c level was 5.5%. Follow-up data from two patients with the *HNF1B* variant showed the need for long-term insulin therapy to control blood glucose levels. One patient (Patient 28) exhibited poor blood glucose levels. During the 3-year follow-up period, the patient had three episodes of DKA, and at the last follow-up, the HbA1c level remained as high as 8.9%. The blood creatinine levels of these patients fluctuated between 90 and 110 µmol/L (normal range 9–88 µmol/L), which were slightly above normal.

### 3.3. Follow-Up Information of Patients With Rare Variants of MODY

Among the six patients carrying the *INS* variant, one was under 2 years of age, and their parents rejected an OGTT examination. The others underwent OGTT to evaluate blood glucose changes and insulin secretion characteristics. Under fasting conditions, blood glucose levels increase, and insulin secretion decreases. Apart from Patient 12, the other four patients had elevated blood sugar levels and no increase in insulin secretion (Figure 3E), which is similar to typical type 1 diabetes mellitus (T1DM) with positive diabetes autoantibody. Therefore, it was easily misdiagnosed as T1DM (Figure 3F). These six patients were followed-up for 1–3 years (Table 3). There were two patients whose insulin doses were downregulated, while the rest had increased doses. In addition, one patient had poor blood glucose control at the last follow-up, despite the increased insulin levels.

Another rare gene identified in our cohort was *ABCC8*. Fasting blood glucose levels were higher than those in patients with *INS* variants, with two out of five patients having fasting C-peptide levels within the normal range and the remaining three having levels below the normal range. Follow-up data were obtained from four patients throughout the duration of 7 months to 3.5 years (Table 3). Three patients (Patients 19, 20 and 21) with the *ABCC8* variant attempted to switch to sulfonylurea therapy after genetic diagnosis; however, they were unsuccessful. Insulin therapy was continued considering the parents' wishes. According to the last follow-up record, the patient's insulin dose was higher than the initial insulin dose. One patient with obesity (Patient 22) had poor blood sugar control even though their insulin usage reached 0.86 U/kg.

## 4. Discussion

Diabetic autoantibodies are important indices for distinguishing T1DM from other types; however, their presence cannot exclude monogenic diabetes mellitus (including MODY). In our cohort, positive autoantibodies were seen in five patients, of which one with the *INS* variant presented as positive for GADA and IA-2A antibodies and the other four (with variants *GCK*, *ABCC8*, *HNF1A* and *INS*, respectively) were positive for a single autoantibody (ICA, IA-2A, GADA and IAA, respectively). Positive autoantibodies to diabetes were also found in common types of MODY (*GCK*- and *HNF4A*/*1A*-MODY) in other studies, whereas the expression of islet cell autoantibodies in patients with MODY is geographically restricted. The positive autoantibody prevalence in individuals with MODY in Great Britain or Japan is reported to be negligible [17, 18]. In the Czech Republic, they reported one quarter of patients with these types (7/28; 25%) was positive for GADA or IA-2A and that GADA was more prevalent (7/7) than IA-2A (1/7) [19]. Similarly, 17% of German and Austrian MODY patients are positive for autoantibodies [20]. The underlying factors for these differences are unknown. Autoantibody-positive MODY patients have been reported to be negative for genetic markers of autoimmunity [21]. This suggests that the transient presence of autoantibodies might be physiologically associated with the process of β-cell destruction that is caused by the primary genetic defects associated with MODY.

In this cohort, DKA mainly occurred in the rare MODY subtypes: *INS* and *ABCC8*. We also identified one patient with *HNF1B*-MODY complicated by DKA. The presence of DKA may be related to the higher glucose levels or poor insulin secretion in our patients. DKA often occurs in patients with T1DM but is rare in MODY; therefore, it is considered as a criterion for differentiating T1DM from MODY. Although DKA is relatively rare, there are relevant reports, observed in some types of MODY, such as *INS*-, *PDX1*-, *NEUROD1*-, *HNF1A*- and *HNF1B*-MODY [11, 22–26]. DKA can also occur in newborn patients with diabetes with the *ABCC8* variant [27]. DKA associated with acute pancreatitis was reported in a 14-year-old patient diagnosed with *ABCC8*-MODY [28]. Exclusion of MODY in presence of DKA as an absolute standard may not be appropriate to, especially for rare types of MODY.

Among the 30 cases of MODY diagnosed at our centre, the most common subtype was *GCK*-MODY (37%), which is consistent with previous domestic and international reports. Among the 11 patients with the *GCK* variant, two had the variant c.571C>T (Het) p.Arg191Trp, and the highest proportion of variants at this locus was found in other MODY cohorts in China, including adults (4/32) [8]. An analysis of Asian *GCK*-MODY cases reported before 2020 found that this locus accounted for the highest proportion of MODY cases in Chinese patients (3/48) [29]; however, due to limited data, it is not yet possible to determine hotspot mutations in the Chinese population.

Surprisingly, in our cohort, six patients with the *INS* variant (20%) were found, ranking secondly. *INS* encodes insulin, which is located at 11p15.5, with three exons. The first exon encodes the ribosome-binding site in mature mRNA; the second encodes the start codon, signal peptide, B-chain and C-peptide; and the third encodes the A-chain. Three of the four variants reported in this study were located in the third exon. Many mutations impair cleavage of the signal peptide and/or proinsulin folding. Misfolded proinsulin is retained in the endoplasmic reticulum (ER), causing ER stress which activates the unfolded protein response and ultimately results in β-cell apoptosis [30]. The novel variant, c.316G>T (Het), forms a truncated protein and may be pathogenic through this mechanism. *INS* variants caused monogenic diabetes in a heterogeneous group of patients. Recessive *INS* variations can cause neonatal diabetes, whereas the dominant *INS* can cause both neonatal diabetes and MODY phenotypes. *INS* variants are the second most common cause of neonatal diabetes in nonconsanguineous families and the rarest cause of MODY [31]. Interestingly, *INS* variants were common in our cohort. Among the five patients with *INS* variant who completed the OGTT, four had similar C-peptide release capacity and blood glucose changes to typical T1DM, and two had 1–2 positive diabetes autoantibodies, which made accurate clinical diagnosis difficult. Without genetic testing, patients are easily misdiagnosed with T1DM [32, 33]. In this study, two patients were positive for diabetes autoantibodies, one younger than 2 years of age and another with a family history of diabetes, for whom the *INS* variant was identified through genetic investigation. For children younger than 2 years, despite diabetes found after 6 months and being positive for diabetes autoantibodies, diabetes related to genetic variants cannot be excluded, thereby validating the necessity to improve genetic investigation for precise diagnosis.

*ABCC8* is another rare MODY-related gene variant with a 17% proportion in our study, which was also reported to be associated with permanent or transient neonatal diabetes mellitus and the opposite phenotype, hyperinsulinaemic hypoglycaemia [34, 35]. The product of *ABCC8* is a sulfonylurea receptor (SUR1), a regulatory subunit of the ATP-sensitive K^+^ channel in membranes of pancreatic β-cells. Activating variants of *ABCC8* can lead to hyperglycaemia. Hyperinsulinaemic hypoglycaemia is usually induced by the inactivation variants in *ABCC8*. In our cohort, there were two patients with insulin secretion function, whereas the rest showed a significant decrease in secretion. One patient (Patient 21) had low insulin secretion and a single positive autoantibody; moreover, their clinical phenotype was similar to that of T1DM. However, since the onset age of the child was less than 2 years, and his mother (who carried the variant) was diagnosed with T1DM at the age of 5 years, genetic testing was conducted. Although most previous reports suggest that *ABCC8*-MODY lacks relevant autoantibodies [36], owing to the heterogeneity of the disease, it is a reminder that clinicians cannot ignore the possibility of genetic variants in younger children with positive autoantibody, especially those with single positive autoantibody.

Genetic diagnosis can help optimize treatment plans for some patients. *GCK*-MODY in childhood does not require special treatment and can be followed up annually. Oral sulfonylurea therapy is particularly effective in patients with *HNF1A*-MODY [37]; however, some patients still required insulin treatment because of the poor efficacy of sulfonylurea in our cohort. This may have been related to a severe decrease in *β*-cell insulin production. Four patients (Patients 13, 14, 16 and 17) with *INS*-MODY experienced an increase in insulin dosage with age, suggesting that the cumulative effect of ER stress was related to the degree of pathological changes due to variants causing further damage to pancreatic islet cells with age. Three patients (Patients 19, 20 and 21) with the *ABCC8* variant attempted to switch to sulfonylurea therapy after genetic diagnosis; however, they were unsuccessful. Insulin therapy continued regarding the wishes of their parents, and the insulin dosage increased with age. Two patients with *HNF1B* variant continued to receive insulin therapy to control blood glucose, with insulin doses slightly lower than those administered to patients with the *ABCC8* variant. In our cohort, some of the classic oral diabetes drugs previously reported to treat MODY were not effective. This reflects the complexity of the clinical phenotype and pathogenesis caused by MODY gene variants; hence, it requires more basic and clinical research to better clarify it.

In conclusion, we found that *GCK*-MODY remained the most common type of MODY at our centre. However, the proportion of the rare *INS* variants was unexpectedly higher than that in previous case series reports and that its clinical phenotype overlapped with that of T1DM; therefore, it can be easily missed. Clinicians should pay close attention to the occurrence of this variant in younger children with suspected type 1 diabetes or children with decreased islet function but negative islet autoantibodies. Genetic testing remains the most important basis for an accurate aetiological diagnosis. In the past, diabetes autoantibody positivity was considered an important indicator to avoid genetic investigations; however, our study suggests that if the patient with positive autoantibody is younger than 2 years old, complete genetic testing should be considered. In addition, DKA is more likely to occur in patients with relatively rarer MODY. And precise genetic diagnosis can help optimize treatment plans, but there is still big challenge in control of blood sugar in some patients because of the complexity of MODY.

## Figures and Tables

**Table 1:** Molecular characteristics of patients diagnosed with MODY.

Patient ID	Gene	DNA change	AA change	Previously reported	Mutation source	Variant classification
1	GCK	c.1343G>T (Het)	p.Gly448Val	Yes	M	LP
2	GCK	c.45+1G>T (Het)	NA	Yes	M	P
3	GCK	c.511T>C (Het)	p.Phe171Leu	Yes	M	LP
4	GCK	c.751A>C (Het)	p.Met251Leu	No	M	LP
5	GCK	c.571C>T (Het)	p.Arg191Trp	Yes	F	P
6	GCK	c.790G>A (Het)	P.Gly264Ser	Yes	F	LP
7	GCK	c.76C>T (Het)	p.Gln26*⁣*^*∗*^	Yes	F	LP
8	GCK	c.127C>T (Het)	p.Arg43Cys	Yes	F	LP
9	GCK	c.769T>C (Het)	p.Trp257Arg	Yes	F	P
10	GCK	c.571C>T (Het)	p.Arg191Trp	Yes	M	P
11	GCK	c.1183G>T (Het)	p.Glu395Ile	Yes	F	LP
12	INS	c.277G>A (Het)	p.Glu93Lys	Yes	De novo	P
13	INS	c.316G>T (Het)	p.Glu106*⁣*^*∗*^	No	De novo	LP
14	INS	c.188-31G>A (Het)	NA	Yes	De novo	P
15	INS	c.250G>A (Het)	p.Gly84Arg	Yes	F	LP
16	INS	c.250G>A (Het)	p.Gly84Arg	Yes	De novo	LP
17	INS	c.188-31(IVS2)G>A (Het)	NA	Yes	De novo	P
18	ABCC8	c.4166T>A (Het)	p.Leu1389Pro	Yes	De novo	LP
19	ABCC8	c.4123G>A (Het)	p.Gly1375Arg	Yes	F	LP
20	ABCC8	c.1176+1G>A (Het)	NA	Yes	F	LP
21	ABCC8	c.695G>A (Het)	p.Trp232*⁣*^*∗*^	Yes	M	LP
22	ABCC8	c.853C>T (Het)	p.Arg285Trp	Yes	F	LP
23	HNF1A	c.802T>A (Het)	p.Phe268Ile	No	F	LP
24	HNF1A	c.1502-2A>G (Het)	NA	Yes	M	P
25	HNF1A	c.34C>T (Het)	p.Leu12Phe	Yes	F	LP
26	HNF1A	c.526+1G>A (Het)	NA	Yes	M	P
27	HNF1A	c.1375_1379delinsTTGC (Het)	p.Gln460Argfs*⁣*^*∗*^25	No	M	P
28	HNF1B	c.494G>A (Het)	p.Arg165His	Yes	De novo	P
29	HNF1B	c.810-2A>G (Het)	NA	No	M	P
30	HNF4A	c.224+2T>G (Het)	NA	No	M	LP

Abbreviations: F, paternal inheritance; Het, heterozygous; LP, likely pathogenic; M, maternal inheritance; P, pathogenic; VUS, uncertain significance.

**Table 2:** Clinical characteristics of the patients diagnosed with MODY.

Characteristic	Total (*N* = 30)	GCK (*N* = 11)	INS (*N* = 6)	ABCC8 (*N* = 5)	HNF4A/1A (*N* = 6)	HNF1B (*N* = 2)	*p* value
Gender: M/F	15/15	6/5	2/4	4/1	1/5	2/0	/
Age at diagnosis (years, mean ± SD)	9.19 ± 4.79	8.05 ± 4.31	7.01 ± 5.32	9.16 ± 6.34	12.47 ± 3.45	12.25 ± 0.35	0.3421
Proportion of family history reports within three generations	63.3%	100%	0	20%	100%	50%	/
BMI (kg/m^2^, mean ± SD)	17.37 ± 3.38	15.63 ± 1.01	15.89 ± 2.64	19.07 ± 4.91	21.07 ± 2.69	16.04 ± 2.63	0.0197
HbA1c (%, mean ± SD)	9.16 ± 3.04	6.62 ± 0.39	11.64 ± 2.86	9.94 ± 2.55	9.10 ± 2.44	14.45 ± 1.77	0.0002
Fasting blood glucose (mmol/L)	8.14 ± 2.95	6.67 ± 0.62	7.90 ± 1.88	10.56 ± 1.43	6.40 ± 1.16	16.15 ± 4.03	0.0022
Fasting C-peptide (nmol/L)	0.39 ± 0.22	0.42 ± 0.13	0.24 ± 0.13	0.37 ± 0.42	0.46 ± 0.15	0.57 ± 0.20	0.0804
Ketoacidosis (%)	4 (13%)	0	1 (17%)	2 (40%)	0	1 (50%)	/
Diabetes autoantibody positivity (%)	5 (17%)	1 (9%)	2 (33%)	1 (20%)	1 (17%)	0	/
1 antibody positive	4	1	1	1	1	0	/
≥2 antibody positive	1	0	1	0	0	0	/
Insulin treatment	12	0	6	4	0	2	/
Metformin treatment	4	0	0	1	3	0	/
Insulin and metformin treatment	1	0	0	0	1	0	/
Sulfonylurea treatment	2	0	0	0	2	0	/
No pharmacological treatment	11	11	0	0	0	0	/

Abbreviations: BMI, body mass index; HbA1C, glycosylated haemoglobin; MODY, maturity-onset diabetes of the young; SD, standard deviation.

**Table 3:** Follow-up results of 10 patients with rare variants.

Genotype	Patient ID	First diagnosis				Last follow-up			
		Age (year)	BMI (kg/m^2^)	Insulin dosage (U/kg)	HbA1c (%)	Age (year)	BMI (kg/m^2^)	Insulin dosage (U/kg)	HbA1c (%)
*INS*	P12	12.09	18.7	0.47	10.8	14.92	19.8	0.21	6.6
	P13	5.42	14.7	0.55	8.3	7.83	15.3	0.9	6.5
	P14	5.50	14.7	0.22	14.5	8.67	15.7	0.63	6.4
	P15	1.33	12.5	1.00	12.0	2.5	14.2	0.73	8.5
	P16	15.08	19.4	0.67	8.9	16	17.5	1.33	11.3
	P17	4.83	15.4	0.45	15.3	7	15.9	0.81	6.8

*ABCC8*	P19	4.17	14.9	0.58	10.6	6.92	14.84	0.7	6.8
	P20	10	16.83	0.59	9.3	13.5	18.12	0.84	8.3
	P21	1.58	15.4	0.45	8.3	5.17	13.54	0.81	6.5
	P22	17.17	26.3	0.94	14	18.25	30.35	0.86	11.3

Abbreviations: BMI, body mass index; HbA1C, glycosylated haemoglobin.

## Data Availability

The data supporting the findings of this study are available from the corresponding author upon reasonable request.

## Supporting Information

Supporting Information Appendix Table S1. Medical histories of the patients. The brief medical histories of 30 patients were compiled in the appendix table, including gender, age at diagnosis, family history of diabetes, BMI, treatment, fasting blood glucose, fasting C-peptide, HbA1c, diabetes autoantibody and the occurrence of DKA.
