# Supplementary material for: Focusing on Rare Variants Related to Maturity-Onset Diabetes of the Young in Children
Source: Pediatr Diabetes. 2025 Jan 28;2025:8155443. doi: 10.1155/pedi/8155443 (PMC12017003; doi:10.1155/pedi/8155443)
Supplement: Supporting Information — Appendix Table S1. Medical histories of the patients. The brief medical histories of 30 patients were compiled in the appendix table, including gender, age at diagnosis, family history of diabetes, BMI, treatment, fasting blood glucose, fasting C-peptide, HbA1c, diabetes autoantibody and the occurrence of DKA. [file 8155443.f1.docx]

Appendix-table 1 Medical histories of the patients

| Patient ID | Type of MODY | Gender | Age at diagnosis（year） | Family history of diabetes | BMI | Treatment | Fasting blood glucose(mmol／L) | Fasting C-peptide(nmol/L) | HbA1c (%) | Diabetes autoantibody（Positive type*） | DKA** |
| --- | --- | --- | --- | --- | --- | --- | --- | --- | --- | --- | --- |
| 1 | GCK | Male | 3.67 | His father was 37 years old and had a fasting blood glucose test of 7.3mmol/L. | 14.30 | No pharmacological treatment | 6.9 | 0.42 | 6.5 | Negative | - |
| 2 | GCK | Male | 3.83 | His mother had GDM and T2DM, and metformin was taken orally. | 14.97 | No pharmacological treatment | 6.8 | 0.47 | 6.3 | Negative | - |
| 3 | GCK | Female | 4.25 | Her mother had GDM. | 16.28 | No pharmacological treatment | 6.5 | 0.34 | 6.4 | Negative | - |
| 4 | GCK | Female | 8.00 | Her mother had GDM and IFG. Her grandmother’s (mother's mother) mother had DM. | 15.10 | No pharmacological treatment | 6.9 | 0.36 | 7.5 | Negative | - |
| 5 | GCK | Male | 14.50 | Father had IFG and grandpa (father's father) had DM | 15.62 | No pharmacological treatment | 7.67 | 0.74 | 6.6 | Negative | - |
| 6 | GCK | Male | 12.75 | When his father was 20 years old, he found that his fasting blood glucose was between 6.3 and 6.7 mmol/L, and he was currently 40 years old. His grandfather, father's sister and grandmother had high fasting blood glucose. | 16.98 | No pharmacological treatment | 6.8 | 0.50 | 6.7 | Negative | - |
| 7 | GCK | Female | 5.50 | Father, grandpa and aunt had high blood glucose, no diagnosis of diabetes and no treatment. | 16.60 | No pharmacological treatment | 6.9 | 0.37 | 6.5 | Negative | - |
| 8 | GCK | Male | 13.75 | Father's fasting blood glucose was around 7.0mmol/L, and currently under drug control, Grandpa was suspected of having high blood glucose. | 16.20 | No pharmacological treatment | 6.1 | 0.34 | 6.5 | positive（ICA） | - |
| 9 | GCK | Male | 6.75 | Grandmother, grandpa, uncle, and aunt had high blood glucose levels, but the details were not available. Father discovered around the age of 27 to 28 that his fasting blood glucose was 6.9 to 7.1 mmol/L, and his 2-hour OGTT blood glucose was 11.3 mmol/L. He was not treated and was followed up regularly. | 15.16 | No pharmacological treatment | 7 | 0.28 | 6.2 | Negative | - |
| 10 | GCK | Female | 4.50 | Her mother suffered from diabetes at the age of 29, with lifestyle conditioning. Her grandfather had diabetes. | 13.99 | No pharmacological treatment | 5.2 | 0.29 | 6.4 | Negative | - |
| 11 | GCK | Female | 11.08 | Her father was 60 years old and had diabetes for 20 years. His grandfather had diabetes for many years. | 16.75 | No pharmacological treatment | 6.6 | 0.46 | 7.2 | Negative | - |
| 12 | INS | Male | 12.00 | No family history of diabetes | 18.70 | Insulin treatment | 6.8 | 0.67 | 10.83 | Negative | - |
| 13 | INS | Female | 4.42 | No family history of diabetes | 14.68 | Insulin treatment | 9.8 | 0.28 | 8.3 | Negative | - |
| 14 | INS | Male | 5.42 | No family history of diabetes | 14.70 | Insulin treatment | 5.4 | 0.17 | 14.5 | Negative | - |
| 15 | INS | Female | 1.33 | No family history of diabetes | 12.50 | Insulin treatment | 10.3 | 0.05 | 12.0 | Positive(ICA, GADA， IA-2A） | + |
| 16 | INS | Female | 3.83 | No family history of diabetes | 19.40 | Insulin treatment | 7 | 0.21 | 8.9 | positive（GADA） | - |
| 17 | INS | Female | 15.08 | No family history of diabetes | 15.36 | Insulin treatment | 8.1mmol/L | 0.28 | 15.3 | Negative | - |
| 18 | ABCC8 | Male | 12.83 | No family history of diabetes | 21.94 | Metformin treatment | 8.9 | 1.09 | 7.5 | Negative | - |
| 19 | ABCC8 | Female | 4.17 | No family history of diabetes | 14.90 | Insulin treatment | 11.3 | 0.14 | 10.6 | Negative | - |
| 20 | ABCC8 | Male | 10.08 | His father's blood glucose was normal at 42 years old, and grandparents had diabetes. | 16.83 | Insulin treatment | 10.6 | 0.12 | 9.3 | Negative | + |
| 21 | ABCC8 | Male | 1.58 | Her mother diagnosed "T1DM" when she was 5 years old | 15.40 | Insulin treatment | 9.5 | 0.09 | 8.3 | positive（IA-2A） | - |
| 22 | ABCC8 | Male | 17.17 | No family history of diabetes | 26.30 | Insulin treatment | 12.5 | 0.43 | 14 | Negative | + |
| 23 | HNF1A | Female | 8.42 | Her father was diagnosed with type 2 diabetes when he was 29 years old. Her grandmother and aunt both suffered from diabetes(without details). | 23.02 | Sulfonylurea treatment | 7.2 | 0.73 | 13.4 | Negative | - |
| 24 | HNF1A | Male | 10.75 | When his father was 38 years old, he found diabetes and was being treated with metformin. Grandpa found diabetes at the age of 45 and was in insulin treatment | 17.48 | Metformin treatment | 5.6 | 0.36 | 7.32 | Negative | - |
| 25 | HNF1A | Female | 12.42 | When her father was 27 years old, he found "type 1 diabetes, myelodysplastic syndrome" and applied insulin therapy | 22.50 | Metformin treatment | 6.22 | 0.37 | 8.4 | Negative | - |
| 26 | HNF1A | Female | 17.83 | Her mother had GDM. At the age of 36, she was diagnosed with diabetes. She was taking betopamine and metformin orally. | 24.43 | Sulfonylurea treatment | 6 | 0.36 | 6.8 | Negative | - |
| 27 | HNF1A | Female | 10.33 | Her mother and grandpa had diabetes. | 20.30 | Insulin and metformin treatment | 5.1 | 0.42 | 8.3 | Positive(IAA) | - |
| 28 | HNF1B | Male | 12.50 | No family history of diabetes | 14.18 | Insulin treatment | 13.3 | 0.71 | 15.7 | Negative | + |
| 29 | HNF1B | Male | 12.00 | His mother was 39 years old and had GDM. Her blood glucose was usually high. | 17.90 | Insulin treatment | 19 | 0.43 | 13.2 | Negative | - |
| 30 | HNF4A | Female | 15.08 | Grandfather had diabetes of unknown type (about 50 years old, controlled by oral antidiabetic drugs) | 18.70 | Insulin and metformin treatment | 8.3 | 0.53 | 10.4 | Negative | - |

*: Anti islet cell antibodies (ICA), Anti insulin antibodies (IAA), Anti glutamic acid decarboxylase antibody (GADA), Protein tyrosine phosphatase antibody (IA-2A)

**:”-“ presented no occurrence, “+” presented occurrence.

IFG：Impaired fasting glucose, IGT: Impaired glucose tolerance, DM: Diabetes mellitus, T2DM: Type 2 diabetes mellitus, T1DM: Type 1 diabetes mellitus, GDM: Gestational diabetes mellitus,
